# Neurological Manifestations of Coronavirus Disease 2019 in Hospitalized Patients: A Lebanese Cohort Study

**DOI:** 10.7759/cureus.35633

**Published:** 2023-03-01

**Authors:** Taghrid El Hajj, Mahmoud Hassoun, Ranime Harb, Oriana Tarabay, Amine Zarzour, Maya Zeineddine

**Affiliations:** 1 Neurology, Rafik Hariri University Hospital, Beirut, LBN; 2 Pulmonary and Critical Care Medicine, Rafik Hariri University Hospital, Beirut, LBN; 3 School of Pharmacy, Lebanese American University, Byblos, LBN; 4 Neurology, Harley Street Medical Center, Abu Dhabi, ARE

**Keywords:** lebanon, middle east, neurological manifestations, sars-cov-2, covid-19, coronavirus

## Abstract

Background: Concerns regarding potential neurologic complications of COVID-19 are being increasingly reported worldwide. Our objective was to investigate the neurologic complications of COVID-19 among a cohort of Lebanese patients with SARS-CoV-2 infection admitted to Rafik Hariri University Hospital (RHUH), the leading COVID-19 testing and treatment center in Lebanon.

Methods: This is a retrospective, single-center, observational study conducted from March to July 2020 at RHUH, Lebanon.

Results: Of 169 hospitalized patients with confirmed SARS-CoV-2 infection (mean {SD} age was 45.75 {19} years and 62.7% were men), 91 patients (53.8%) had severe infection and 78 patients (46.2%) had non-severe infection according to the American Thoracic Society guidelines for community-acquired pneumonia. Overall, 112 patients (66.3%) developed neurologic symptoms: CNS (46.1%), PNS (43.7%), and skeletal muscle injury (2.4%). Compared with patients with non-severe infection, patients with severe infection were significantly older, were male and more likely to have underlying disorders, especially diabetes and cardiac or cerebrovascular disease. Moreover, those patients experienced more typical COVID-19 symptoms at onset of illness such as fever, cough and fatigue. However, there was no significant difference in the frequency of all nervous system manifestations between the severe and the non-severe infection groups (57 {62.6%} vs 55 {70.5%}; p =0.316), except for impaired consciousness, where seven patients had impaired consciousness in the severe group compared to none in the non-severe group (p=0.012).

Conclusion: A wide variety of neurologic symptoms were detected in our Lebanese cohort of hospitalized COVID-19 patients. A comprehensive knowledge of the neurologic manifestations will help healthcare providers to be more attentive to these complications.

## Introduction

Coronavirus disease 2019 (COVID-19), the disease caused by severe acute respiratory syndrome coronavirus type 2 (SARS-CoV-2), is an emerging infectious disease, with the first cases identified in Wuhan, China, in December 2019 [1-2]. As the virus has continued to spread since then, COVID-19 was defined as a pandemic by the World Health Organization (WHO) in March 2020. Until February 22, 2022, there were 427,828,520 confirmed cases of COVID-19 and 5,922,484 deaths globally. In Lebanon, a total of 1,053,252 cases of COVID-19 and 10,007 deaths were recorded [3].

Although common manifestations of the disease include respiratory tract and associated systemic manifestations, neurologic manifestations are being increasingly recognized. Initially, the neurologic complications were alerted to the high prevalence of anosmia and dysgeusia, which occur in early phases of COVID-19 disease and in the absence of other clinical features [4]. Moreover, early cohorts featured non-specific symptoms such as headache and dizziness [5]. However, a growing number of case reports and series describing a wide array of severe neurological manifestations are emerging, including central nervous system (CNS) dysfunction, altered level of consciousness, focal complications (stroke, encephalitis, or seizures), peripheral nervous system (PNS) and skeletal muscle complications like myopathy [6-11], and autoimmune manifestations such as Guillain-Barre syndrome [12].

Neurotropism is one common feature of previously identified coronaviruses such as severe acute respiratory syndrome coronavirus and Middle East respiratory syndrome coronavirus, as well as H1N1 influenza A virus [13]. It has been suggested that the involvement of the nervous system can be either during the acute phase of the infection, thought to reflect direct action of these viruses on the nervous tissue, or later as post-infectious, probably due to an indirect action through the activation of immune-mediated mechanisms [13]. Coronaviruses could reach the CNS via circulation or across the cribriform plate of the ethmoid. Endothelium, glial cells, and neurons have been reported to express angiotensin-converting enzyme receptor 2 (ACE 2), the receptor through which SARS-CoV-2 virus enters the cells [14].

However, to our knowledge, there were no studies describing the neurologic manifestations of COVID-19 in the Lebanese population. Therefore, we report the neurologic symptoms of COVID-19 among a cohort of Lebanese patients with SARS-CoV-2 infection admitted to Rafik Hariri University Hospital (RHUH), the leading COVID-19 testing and treatment center in Lebanon in 2020.

## Materials and methods

Study design and participants

This is a retrospective, single-center, observational study conducted at Rafik Hariri University Hospital, a governmental hospital assigned by the Ministry of Public Health to treat patients with COVID-19 in Beirut, Lebanon. During the exponential phase of the pandemic in Lebanon, we reviewed the medical records of all patients admitted to our hospital from March to July 2020 and who had been diagnosed with COVID-19. All patients included in this study had a confirmed laboratory diagnosis of COVID-19, either through a positive real-time reverse transcription-polymerase chain reaction (rt-PCR) of throat or swab samples or a positive blood test for immunoglobulin G (IgG)/immunoglobulin M (IgM) antibodies against SARS-CoV-2.

Data collection

Electronic medical records and peak laboratory parameters, performed at the time of COVID-19 diagnosis and/or anytime during hospitalization, were reviewed for all patients with confirmed SARS-CoV-2 infection. Demographic data such as age, sex, previous comorbidities (hypertension, diabetes, cardiac or cerebrovascular disease, neuropsychiatric disease, respiratory disease, thyroid disorders, malignancy, and chronic kidney disease); typical COVID-19 symptoms from onset to hospital admission (fever, cough, fatigue, rhinorrhea, diarrhea, throat pain, abdominal pain, conjunctivitis); nervous system symptoms; and laboratory findings were recorded. Any missing or uncertain records were clarified through direct communication with involved patients, their family members, and treating physician. Severity of COVID-19 (mild versus moderate versus severe) was defined according to the 2007 Infectious Diseases Society of America/American Thoracic Society guidelines for community-acquired pneumonia [15]. Patients with moderate and severe infection were classified as “Severe” and patients with mild infection were labeled as “Non-severe”.

All neurologic manifestations were extracted and categorized into three categories: CNS manifestations (dizziness, headache, ataxia, impaired consciousness, acute cerebrovascular disease including ischemic stroke and intracerebral hemorrhage); PNS manifestations (taste impairment, smell impairment, vision impairment and nerve pain); and skeletal muscular injury manifestations defined as muscle pain associated with an elevated serum creatine kinase level greater than 200 U/L [16]. For this study, obtaining written informed consent from the participants was waived given that all data was collected retrospectively and anonymously.

Statistical analysis

Statistical analyses were performed using IBM SPSS Version 24 (IBM Corp, Armonk, NY, USA). The clinical characteristics of patients with COVID-19 were summarized using descriptive statistics, which included frequency counts and percentages, while continuous variables were expressed as mean ± standard deviation (SD). Laboratory parameters evaluated were expressed as range (minimum-maximum). Differences between severe and non-severe clinical conditions for sex, comorbidities, typical symptoms, and nervous system symptoms were tested using Pearson Chi-square, while for age, the independent t test was used. The Mann-Whitney U test was performed to compare laboratory findings. All analyzes were carried out at 0.05 significance level.

## Results

Demographic and clinical characteristics

A total of 169 hospitalized patients with confirmed SARS-CoV-2 infection were identified and included in this study. Their demographic and clinical characteristics are presented in Table 1. The mean (SD) age was 45.75 (19) years and 62.7% were men. Of these patients, 60 (35.5%) had at least one underlying disease. The most common systemic comorbidities were hypertension (18.9%), cardiac or cardiovascular diseases (10.7%), and diabetes (10.1%). The most common COVID-19 symptoms upon admission were: cough (47.9%), fever (46.2%), rhinorrhea (12.4%), and throat pain (12.4%). One hundred and twelve patients (66.3%) developed neurologic symptoms: CNS (46.1%), peripheral nervous system (PNS) (43.7%), and skeletal muscle injury (2.4%). One patient had intractable hiccups, which could be classified as either peripheral or central nervous system manifestation. Among patients with CNS features, the most commonly reported symptom was headache (41.4%). In patients with PNS manifestations, the most common recorded symptoms were taste (37.3%) and smell (36.1%) impairment. The neurologic manifestations occurred early in the illness (mean time = two days).

**Table 1:** Clinical characteristics of patients with coronavirus disease 2019 (COVID-19) * P values indicate differences between patients with moderately severe to mild infection. A P-value less than 0.05 was considered statistically significant. SD=Standard Deviation

Characteristic	Total (n=169)	Severe (n=91)	Non-severe (n=78)	P-value*
Age, mean (SD), years	45.75 ± 19	53.8 ± 18	36.3 ± 15	<0.001
Age, years				
<50	95 (56.2%)	36 (39.6%)	59 (75.6%)	
≥50	74 (43.8%)	55 (60.4%)	19 (24.4%)	<0.001
Sex				
Female	63 (37.3%)	26 (28.6%)	37 (47.4%)	
Male	106 (62.7%)	65 (71.4%)	41 (52.6%)	0.011
Comorbidities				
Any	60 (35.5%)	40 (44.0%)	20 (25.6%)	0.013
Hypertension	32 (18.9%)	21 (23.1%)	11 (14.1%)	0.138
Diabetes	17 (10.1%)	15 (16.5%)	2 (2.6%)	0.003
Cardiac or Cardiovascular disease	18 (10.7%)	14 (15.4%)	4 (5.1%)	0.031
Respiratory disease	6 (3.6%)	5 (5.5%)	1 (1.3%)	0.140
Neuro-Psychiatric disease	5 (3.0%)	1 (1.1%)	4 (5.1%)	.202
Thyroid Disorders	3 (1.8%)	2 (2.2%)	1 (1.3%)	.625
Malignancy	3 (1.8%)	3 (3.3%)	0	.250
Chronic Kidney Disease	1 (0.6%)	0	1 (1.3%)	.462
Typical Symptoms				
Any	130 (76.9%)	90 (98.9%)	40 (51.3%)	<0.001
Fever	78 (46.2%)	54 (59.3%)	24 (30.8%)	<0.001
Cough	81 (47.9%)	61 (67.0%)	20 (25.7%)	<0.001
Rhinorrhea	21 (12.4%)	10 (11.0%)	11 (14.1%)	0.541
Fatigue	17 (10.1%)	15 (16.5%)	3 (3.8%)	0.008
Throat pain	21 (12.4%)	11 (12.1%)	10 (10.8%)	0.886
Diarrhea	12 (7.1%)	6 (6.6%)	6 (7.7%)	0.782
Abdominal pain	2 (1.2%)	1 (1.1%)	1 (1.3%)	0.913
Conjunctivitis	1 (0.6%)	0	1 (1.3%)	0.279
Nervous System Symptoms				
Any	112 (66.3%)	57 (62.6%)	55 (70.5%)	0.316
Headache	70 (41.4%)	36 (39.6%)	34 (43.6%)	0.596
Dizziness	9 (5.3%)	6 (6.6%)	3 (3.8%)	0.399
Ataxia	1 (0.6%)	1 (1.1%)	0	0.345
Impaired Consciousness	7 (4.1%)	7 (7.7%)	0	0.012
Seizures	0	0	0	--
Acute Cerebrovascular disease	0	0	0	--
Myalgia	43 (25.4%)	23 (25.3%)	20 (25.6%)	0.942
Impairment				
Taste	63 (37.3%)	29 (31.9%)	34 (43.6%)	0.159
Smell	61 (36.1%)	28 (30.8%)	33 (42.3%)	0.162
Vision	2 (1.2%)	0	2 (2.6%)	0.131
Hearing	0	0	0	--
Speech	0	1	0	0.335
Nerve Pain	8 (4.7%)	3 (3.3%)	5 (6.4%)	0.368
Skeletal Muscle Pain/Injury	4 (2.4%)	2 (2.25)	2 (2.6%)	0.912
Intractable Hiccups	1 (0.6%)	0	1 (1.3%)	0.289
Onset of any neurological symptoms to hospital admission, mean (range), days	2 (1-11)	2 (1-11)	2 (1-10)	0.284

In our Lebanese cohort, 91 patients (53.8%) had severe infection and 78 patients (46.2%) had non-severe infection according to the American Thoracic Society guidelines for community-acquired pneumonia [15] (Appendix 1). The patients with severe infection were significantly older (mean {SD} age, 53.8 {18} years vs 36.3 {15} years; p <0.001), were male (65 {71.4%} vs 41 {52.6%}; p =0.011) and more likely to have comorbidities (40 {44.0%} vs 20 {25.6%}; p= 0.013), especially diabetes (15 {16.5%} vs 2 {2.6%}; p=0.003) and cardiac or cerebrovascular disease (14 {15.4%} vs 4 {5.1%}; p=0.031). Moreover, those patients experienced more typical COVID-19 symptoms at onset of illness (90 {98.9%} vs 40 {51.3%}; p<0.001), especially fever (54 {59.3%} vs 24 {30.8%}; p<0.001), cough (61 {67.0%} vs 20 {25.7%}; p<0.001) and fatigue (15 {16.5%} vs 3 {3.8%}; p<0.008).

However, there was no significant difference in the frequency of all nervous system manifestations between the severe and the non-severe infection groups (57 {62.6%} vs 55 {70.5%}; p =0.316), except for impaired consciousness, where 7 patients had impaired consciousness in the severe group compared to none in the non-severe group (p=0.012).

Laboratory findings in patients with severe and non-severe infection

The laboratory findings in severe and non-severe subgroups were presented in Table 2. Patients with severe infection tended to have increased inflammatory markers with a significantly higher C-reactive protein level (CRP) compared with those patients with non-severe infection (median, 19.9 mg/L {range, 0.1 - 500} vs 3.5 mg/L {range, 0.1 - 190}; p<0.001). Furthermore, severely infected patients had significantly elevated aspartate aminotransferase levels (median, 33 U/L {range, 13 - 10762} vs 22 U/L {range, 13 - 85}; p=0.004), blood urea nitrogen level (BUN) (median, 13 mmol/L {range, 5 - 151} vs 11 mmol/L {range, 5 - 35}; p<0.001) and creatinine level (median, 0.85 µmol/L {0.36 - 6.5} vs 0.78 µmol/L {0.5 - 1.3}; p=0.044), suggesting multiple organ damage. Surprisingly, creatinine kinase (CPK), lactate dehydrogenase (LDH) and D-dimer levels did not significantly differ between the two groups (severe vs non-severe). 

**Table 2:** Laboratory findings of patients with coronavirus disease 2019 (COVID-19) * P values indicate differences between patients with moderately severe to mild infection. P-value less than 0.05 was considered statistically significant. L=Liter; mg/L=milligrams per liter; U/L=Units per liter; SGPT=serum glutamic-pyruvic transaminase; SGOT=serum glutamic-oxaloacetic transaminase; mmol/L=millimoles per liter; µmol/L=micromoles per liter

Laboratory Finding	Total Median (range)	Severe Median (range)	Non-severe Median (range)	P-value
Count, x 10^9^/L (n=149)				
White blood cell	6.0 (2.18 – 17.5)	5.8 (2.18 - 14.1)	6.1 (2.88 - 17.5)	0.912
Neutrophil	2.7 (0.1 – 14.3)	2.8 (0.1 - 12.8)	2.7 (0.5 - 14.3)	0.170
Lymphocyte	1.6 (0.1 - 9.4)	1.5 (0.1 - 9.4)	1.7 (0.3 - 6.0)	0.182
Platelet	207 (22 – 483)	204 (22 - 483)	207 (102 - 425)	0.657
C-reactive Protein, mg/L (n=137)	9.0 (0.1-500)	19.9 (0.1 - 500)	3.5 (0.1 - 190)	<0.001
Creatinine Kinase, U/L (n=38)	90.5 (16 – 17354)	90.5 (38 -17354)	103 (16 - 222)	0.883
D-dimer, mg/L (n=16)	0.67 (0.01-19)	0.67 (0.01 - 19)	1.02 (0.18 - 1.86)	0.817
Lactate Dehydrogenase, U/L (n=54)	242.5 (36.5 – 5102)	250 (36.5 – 5102)	234 (166 – 783)	0.269
Aminotransferase, U/L				
SGPT (n=100)	23 (9 – 5476)	24 (10 - 5476)	22 (9 - 109)	0.125
SGOT (n=87)	27 (13 – 10762)	33 (13 - 10762)	22 (13 – 85)	0.004
Blood Urea Nitrogen, mmol/L (n=136)	12 (5 - 151)	13 (5 - 151)	11 (5 - 35)	<0.001
Creatinine,µmol/L (n=145)	0.83 (0.36 – 6.5)	0.85 (0.36 – 6.5)	0.78 (0.5 – 1.3)	0.044

Laboratory findings in patients with and without neurologic symptoms 

Laboratory findings of patients with and without neurologic manifestations were tabulated in Table 3. Patients with neurologic symptoms had a significantly lower neutrophil count (median, 2.3 x 10^9^/L {range, 0.10-8.70} vs 3.5 x 10^9^/L {range, 0.50-14.3}; p=0.028). In the severe group, patients with neurologic symptoms had higher lymphocytic count (Lymphocytic Count, median, 1.8 x 10^9^/L {range, 0.10-8.30} vs 1.1 x 10^9^/L {range, 0.20-9.4} than patients without neurologic symptoms; p=0.028). There were no statistically significant differences in all the other laboratory markers of patients with or without neurologic symptoms in both groups.

**Table 3:** Laboratory findings of patients with COVID-19 with and without neurological symptoms * P values indicate differences between patients with moderately severe to mild infection. P-value less than 0.05 was considered statistically significant. COVID-19=Coronavirus Disease 2019; CPK= Creatinine Kinas; WBC=White Blood Cell; CRP=C-Reactive Protein; SGOT=serum glutamic-oxaloacetic transaminase; SGPT=serum glutamic-pyruvic transaminase; LDH=Lactate Dehydrogenase; BUN=Blood Urea Nitrogen; U/L=Units per liter; L=Liter; mg/L=milligrams per liter; µmol/L=micromoles per liter; mmol/L=millimoles per liter

	Median (range)								
	Total			Severe			Non Severe		
Laboratory Findings	With Neurologic Symptoms (N = 112)	Without Neurologic Symptoms (N = 57)	p-value	With Neurologic Symptoms (N = 57)	Without Neurologic Symptoms (N = 34)	p-value	With Neurologic Symptoms (N = 55)	Without Neurologic Symptoms (N = 23)	p-value
CPK, U/L	84.50 (38.00 – 435.00)	96.50 (16.00 – 17354.00)	.976	95.50 (38.00 – 435.00)	90.50 (41.00 – 17354.00)	.737	83.00 (44.00 – 222.00)	148.50 (16.00 – 191.00)	.670
WBC, x 10^9^/L	5.94 (2.18 – 12.60)	6.47 (2.88 – 17.50)	.179	5.99 (2.18 – 10.00)	5.70 (2.88 – 14.10)	.670	5.90 (2.88 – 12.60)	7.21 (3.20 – 17.50)	.062
Lymphocytes, x 10^9^/L	1.80 (0.10 – 8.30)	1.50 (0.20 – 9.40)	.122	1.80 (0.10 – 8.30)	1.10 (0.20 – 9.40)	.065	1.70 (0.30 – 6.00)	1.70 (0.80 – 3.60)	.889
Neutrophils, x 10^9^/L	2.30 (0.10 – 8.70)	3.50 (0.50 – 14.30)	.028	2.30 (0.10 – 8.70)	2.80 (0.50 – 12.80)	.365	2.30 (0.50 – 6.30)	3.65 (0.70 – 14.30)	.017
Platelets, x 10^9^/L	198.50 (22.00 – 473.00)	207.00 (120.00 – 483.00)	.531	208.00 (22.00 – 473.00)	201.00 (120.00 – 483.00)	.652	194.00 (102.00 – 425.00)	230.00 (147.00 – 339.00)	.114
CRP, mg/L	8.45 (0.10 – 282.00)	16.00 (0.10 – 500.00)	.113	12.70 (0.10 – 282.00)	28.00 (0.90 – 500.00)	.152	3.40 (0.10 – 47.90)	4.25 (0.10 – 190.00)	.759
SGOT, U/L	25.00 (13.00 – 137.00)	32.00 (13.00 – 10762.00)	.295	31.00 (13.00 – 137.00)	33.50 (13.00 – 10762.00)	.503	22.00 (13.00 – 59.00)	22.00 (13.00 – 85.00)	.750
SGPT, U/L	24.00 (10.00 – 212.00)	19.00 (9.00 – 5476.00)	.359	25.00 (11.00 – 212.00)	21.50 (10.00 – 5476.00)	.981	23.00 (10.00 – 109.00)	16.00 (9.00 – 39.00)	.108
D-Dimer, mg/L	0.40 (0.01 – 2.74)	0.74 (0.30 – 19.00)	.222	0.67 (0.01 – 2.74)	0.74 (0.30 – 19.00)	.473	0.18 (0.18 – 0.18)	1.14 (0.42 – 1.86)	.221
LDH, U/L	237.00 (36.50 – 979.00)	292.00 (168.00 – 5102.00)	.176	237.00 (36.50 – 979.00)	328.00 (168.00 – 5102.00)	.137	237.00 (166.00 – 388.00)	217.00 (169.00 – 783.00)	.933
Creatinine, µmol/L	0.85 (0.36 – 6.50)	0.82 (0.50 – 6.40)	.893	0.89 (0.36 – 6.50)	0.82 (0.56 – 6.40)	.992	0.80 (0.50 – 1.30)	0.70 (0.50 – 1.30)	.485
BUN, mmol/L	12.00 (6.00 – 70.00)	12.00 (5.00 – 151.00)	.598	12.50 (7.00 – 70.00)	14.00 (5.00 – 151.00)	.367	11.00 (6.00 – 27.00)	11.00 (5.00 – 35.00)	.579

## Discussion

To our knowledge, this is the first report on the neurological manifestations of hospitalized Lebanese patients with COVID-19. Out of 169 patients included in this study, 91 (53.8%) had severe infection and 78 (46.2%) had non-severe infection. A total of 112 (66.3%) had various neurologic manifestations that involved CNS, PNS and to a lesser extent, skeletal muscles, highlighting the high incidence of neurologic symptoms among patients with SARS-CoV-2 infection. In general, the most common neurologic manifestations observed in our cohort were headache (41.4%), taste (37.3%) and smell impairment (36.1%). Compared with patients with non-severe infection, patients with severe infection were older, males and had more comorbidities, mainly diabetes and cardiac or cerebrovascular diseases. This finding is consistent with previous studies showing that older age, male sex, and presence of comorbid cardiac disease or diabetes were important predictors of severe illness [6,17-19].

Moreover, patients with severe infection experienced more typical symptoms such as fever, cough, and fatigue. This is in contrast to the Wuhan study, where patients with severe infection had fewer typical symptoms [6].

However, unlike previous studies, which showed a tendency for patients with severe infection to develop neurologic manifestations [6,11], our study did not show any significant difference in the frequency of all nervous system manifestations between the severe and the non-severe infection groups, except for impaired consciousness where seven patients had impaired consciousness in the severe group compared to none in the non-severe group (p=0.012). This could be due to the fact that less attention was given to the neurological symptoms in patients with severe COVID-19 disease because of the predominance of respiratory symptoms and the limited ability to report neurological symptoms in severely ill patients. Nevertheless, other studies showed that loss of smell and taste were associated with lower mortality rate and less severe course of the disease [20-21]. 

Moreover, during the early epidemic period of COVID-19, neurologic symptoms, except for anosmia and dysgeusia, were not increasingly recognized as complications of COVID-19 infection. Furthermore, in our cohort, most neurologic manifestations occurred early in the illness, with a mean time of two days. However, some patients without typical COVID-19 symptoms presented to the hospital with only neurologic manifestation as their initial complaint. This highlights (1) the need to pay closer attention to neurologic symptoms, especially for patients with severe infections, and (2) always consider SARS-CoV-2 infection as a differential diagnosis in patients presenting with only neurologic manifestations to avoid delay in diagnosis or misdiagnosis and thus, prevent the transmission of the infection and improve the patients’ outcomes.

Patients with severe infection had increased inflammatory markers with a significantly higher CRP compared with those patients with non-severe infection. Moreover, severely infected patients had significantly elevated aspartate aminotransferase levels, BUN and creatinine level, suggesting multiple organ damage. Nevertheless, unlike the Wuhan Study [6], we did not detect significant differences between levels of CPK, LDH, and D-dimer between two groups (severe vs non-severe). Similarly, there were no statistically significant differences in all the laboratory markers of patients with or without neurologic symptoms in both groups, except for a lower neutrophil count in patients with neurologic symptoms. This could be explained by the fact that those laboratory tests were only performed on a small number of patients due to their high cost and limited feasibility during the epidemic.

Neurologic manifestations in the setting of COVID-19 can be explained by direct invasion of the virus to the nervous system or by “cytokine storm” which triggers inflammatory responses [22]. In fact, any damage in the neurons or glial cells can lead to neurologic pathologies [23]. Angiotensin-converting enzyme 2 (ACE2) was identified on January 2020 [24] as the functional receptor for SARS-CoV-2, which is found in multiple human organs, including nervous system and skeletal muscles [25]. Coronaviruses are able to directly attack the brain via the viral S protein that can bind to the ACE2 receptor. The cell tropism of the virus is defined by the expression of the ACE2 receptor in the cell. Autopsy analyses among 17 COVID-19 patients highlighted that 47% of the patients had SARS-CoV-2 positive in brain tissue with cerebral edema and vascular congestion [26]. Similar brain autopsy results of patients with COVID-19 were reported in the literature suggesting that ACE2 receptors are widely expressed in neurons, astrocytes, and oligodendrocytes [22]. All these findings point toward a molecular mechanism by which SARS-CoV-2 invades the brain and then causes neuronal injury. Furthermore, the multi-organ damage in some patients with COVID-19 as well as the damage in the brain can be explained by another hypothesis, now labeled “cytokine storm”. Actually, the release of a large number of pro-inflammatory cytokines enhances the permeability of the vessels, abnormal clotting, and multiple organ failure. This cytokine storm hypothesis is reflected in our study by the higher CRP, creatinine, and Serum Glutamic Oxaloacetic Transaminase (SGOT) levels in the severe group, suggesting damage to multiple organs, including kidneys and liver. On the other hand, encephalopathy due to hypoxia or systemic inflammation seems to be the most common CNS complication of COVID-19 [27]. Risk factors for encephalopathy include older or immunosuppressed patients with cardiovascular, hepatic and renal comorbidities [6]. In our cohort, we identified seven patients with depressed level of consciousness in the severe group versus none in the non-severe group, showing an increased risk of encephalopathy in patients with severe COVID-19 disease. Moreover, cerebrovascular disease is also identified as one of the main SARS-CoV-2 neurological complication. The pooled incidence of COVID-19 related to acute cerebrovascular disease is concluded as 2.3% [28]. In fact, brain autopsies of hospitalized patients with COVID-19 who were diagnosed with ischemic or hemorrhagic stroke showed endothelium injury and thrombotic microangiography rather necrotizing encephalitis or vasculitis [29]. Several studies have demonstrated that the “cytokine storm” may stimulate the formation of microthrombi by activating the coagulation system [22]. This evidenced that severely infected patients are more likely to develop cerebrovascular manifestations due to coagulopathy [30]. Surprisingly, in our study, we could not identify any patient with cerebrovascular disease and this is mainly due to the fact that brain imaging could not be performed in patients with neurologic symptoms and even in patients with impaired consciousness in whom there is a high chance to detect ischemic or hemorrhagic brain lesions.

Inconsistent with previous studies, skeletal muscle injury was found to be rare in our study with no significant difference between the severe and the non-severe groups [6,16, 31-32]. This could be explained by the fact that CPK was performed in only a small number of patients. Skeletal muscle injury could be due to ACE2 expression in skeletal muscle [33]. Though, in postmortem analysis, SARS-CoV-2, using the same receptor, was not identified in skeletal muscle [34]. Therefore, whether SARS-Cov-2 attacks skeletal muscle cells by binding to ACE2 receptors needs to be further inspected.

Our study has several limitations. First of all, the sample size of 169 patients was too small to allow significant differences between the severe and non-severe groups. Second, the data was obtained retrospectively from medical records, so selection bias may arise and some important information could be missing. Certain patients with mild neurologic symptoms might not be captured. The main limitation of this work is the ever-present pandemic context and the influx of a big number of patients infected with SARS-Cov-2 infection to RHUH, which prevented us from performing a full neurological evaluation and a complete diagnostic work up. In fact, magnetic resonance imaging, lumbar puncture and nerve conduction studies were eluded to decrease the risk of cross infection. Therefore, this study is mainly descriptive and cannot conclude if the neurologic problems of our hospitalized patients were caused by the SARS-Cov-2 infection itself or by other factors such as cross-immunity, inflammatory reaction, or adverse events of one of the treatments. Finally, it is a hospital-based study, thus, it does not mirror the incidence of neurologic manifestations in the outpatients with COVID-19 and its findings cannot be generalized to the patients in the community.

## Conclusions

In conclusion, COVID-19 was commonly associated with neurologic manifestations. A wide variety of neurologic symptoms were detected in our Lebanese cohort of hospitalized COVID-19 patients, which is probably related to different pathologic pathways, highlighting the need for neurologists to be included in the COVID-19 response team routinely for early diagnosis and management of SARS-CoV-2 neurologic complications in order to improve patients’ outcomes. However, larger sample studies with full diagnostic workup, which includes brain imaging, cerebrospinal fluid analysis, and objective peripheral nervous system evaluation, are needed to characterize the neurologic involvement in SARS-CoV2 infected patients better and predict their outcome. Furthermore, the mechanisms of neurologic injury and their long-term significances need to be further studied in future research.

## Appendices

**Table 4:** Infectious Diseases Society of America/American Thoracic Society Criteria for Defining Severe Community-acquired Pneumonia PaO2= Partial pressure of oxygen, FiO2= Fraction of inspired oxygen, Min= minutes; mg/dl= milligrams per deciliters; μl= microliters; °C = degree Celsius

Validated definition includes either one major criterion or three or more minor criteria
Minor criteria
Respiratory rate ≥ 30 breaths/min
Pa_O2_/Fi_O2_ ratio ≤ 250
Multilobar infiltrates
Confusion/disorientation
Uremia (blood urea nitrogen level ≥ 20 mg/dl)
Leukopenia due to infection alone (white blood cell count
Thrombocytopenia (platelet count
Hypothermia (core temperature
Hypotension requiring aggressive fluid resuscitation
Major criteria
Septic shock with need for vasopressors
Respiratory failure requiring mechanical ventilation
